# Elevated caudate connectivity in cognitively normal Parkinson’s disease patients

**DOI:** 10.1038/s41598-020-75008-6

**Published:** 2020-10-21

**Authors:** Natalie Wright, Abrar Alhindi, Colleen Millikin, Mandana Modirrousta, Sean Udow, Andrew Borys, Julius Anang, Douglas E. Hobson, Ji Hyun Ko

**Affiliations:** 1 Department of Human Anatomy and Cell Science, Max Rady College of Medicine, Rady Faculty of Health Sciences, University of Manitoba, Winnipeg, MB R3E 0J9 Canada; 2 Neuroscience Research Program, Kleysen Institute for Advanced Medicine, Health Sciences Centre, Winnipeg, MB R3E 3J7 Canada; 3 Department of Clinical Health Psychology, Max Rady College of Medicine, Rady Faculty of Health Sciences, University of Manitoba, Winnipeg, MB R3E 3N4 Canada; 4 Department of Psychiatry, Max Rady College of Medicine, Rady Faculty of Health Sciences, University of Manitoba, Winnipeg, MB R3T 2N2 Canada; 5 Section of Neurology, Department of Internal Medicine, Max Rady College of Medicine, Rady Faculty of Health Sciences, University of Manitoba, Winnipeg, MB R3A 1R9 Canada; 6 St. Boniface Clinic, Winnipeg, MB R2H 2A5 Canada; 7 Present Address: Human Anatomy Department, College of Medicine, King Abdulaziz University, Jeddah, 21589 Saudi Arabia

**Keywords:** Cognitive neuroscience, Computational neuroscience, Parkinson's disease, Neurodegenerative diseases, Biomarkers, Brain imaging, Magnetic resonance imaging

## Abstract

Mild cognitive impairment (MCI) is common in Parkinson’s disease patients. However, its underlying mechanism is not well understood, which has hindered new treatment discoveries specific to MCI. The aim of this study was to investigate functional connectivity changes of the caudate nucleus in cognitively impaired Parkinson’s patients. We recruited 18 Parkinson’s disease patients—10 PDNC [normal cognition Parkinson’s disease; Montreal Cognitive Assessment (MoCA) ≥ 26], 8 PDLC (low cognition Parkinson’s disease; MoCA < 26) —and 10 age-matched healthy controls. All subjects were scanned with resting-state functional magnetic resonance imaging (MRI) and perfusion MRI. We analyzed these data for graph theory metrics and Alzheimer’s disease-like pattern score, respectively. A strong positive correlation was found between the functional connectivity of the right caudate nucleus and MoCA scores in Parkinson’s patient groups, but not in healthy control subjects. Interestingly, PDNC’s functional connectivity of the right caudate was significantly higher than both PDLC and healthy controls, while PDLC and healthy controls were not significantly different from each other. We found that Alzheimer’s disease-like metabolic/perfusion pattern score correlated with MoCA scores in healthy controls, but not in Parkinson’s disease. Increased caudate connectivity may be related to a compensatory mechanism found in cognitively normal patients with Parkinson’s disease. Our findings support and complement the dual syndrome hypothesis.

## Introduction

Parkinson’s disease is traditionally thought of as a movement disorder, but the majority of patients have varying degrees of cognitive impairment. These range from mild cognitive impairment (MCI) to severe dementia^1^. MCI can present at time of diagnosis in about 35% of cases, and it has been estimated that about 62% of those patients progress to dementia over 5 years^2^. However, reconversion from MCI to normal cognition is not uncommon (~ 20% at one-year follow up^3^). Cognitive impairment in Parkinson’s disease affects several cognitive domains, such as executive function, attention, visuospatial, memory and language^1^. Clinical presentation of cognitive impairment therefore varies. Some MCI patients experience deficits in working memory and executive function^4^; others are more impaired in visuospatial function^5^. Several possible mechanisms have been proposed for cognitive impairment in Parkinson’s disease, including dopamine depletion in the frontostriatal pathway^6^, and imbalance in neurotransmitters, such as acetylcholine^7^, serotonin^8–10^, and norepinephrine^11^. The presence of cortical Lewy bodies in the frontal cortex could also contribute to this impairment^12^. Response to cholinergic medications has been reported to be different among cognitively impaired Parkinson’s patients: while Parkinson’s disease dementia is routinely treated with cholinesterase inhibitors, these are not consistently used in patients with MCI^13^.

This heterogeneity has led to the emergence of the dual syndrome hypothesis^14^. This hypothesis differentiates cognitively impaired Parkinson’s disease patients into two groups. The first group: (i) tremor-dominant patients with MCI, who experience impairment in executive functions. This is thought to be due to dysfunction in the frontostriatal network, involving the caudate nucleus and prefrontal cortex. The second group posited by dual syndrome hypothesis: (ii) patients with early postural instability and gait imbalance, expressing more visuospatial dysfunction due to posterior cortical and temporal lobe dysfunction (symptomatically similar to what is found in Alzheimer’s disease). These patients progress rapidly to dementia, and respond relatively well to acetylcholinesterase inhibitors, the most often prescribed medications for dementia associated with Alzheimer’s disease.

While some studies align with the dual syndrome hypothesis^15–17^, others suggest that frontal lobe dysfunction is also relevant to dementia development. For example, longitudinal studies have repeatedly reported that frontal executive function is a strong predictor for later dementia development along with posterior-visuospatial dysfunction^18–21^.

Adopted from the dual syndrome hypothesis^14^, we viewed the frontal executive dysfunction as Parkinson’s disease-specific, and posterior visuospatial dysfunction as Alzheimer’s disease-like cognitive deficits in Parkinson’s disease^22^. To quantitate the level of progression of Parkinson’s disease-specific and Alzheimer’s disease-like pathology within each individual, we have used two different functional magnetic resonance imaging (fMRI) approaches, i.e., resting-state functional connectivity analysis and pseudo-continuous arterial spin labeling (pCASL), respectively. Graph theory analysis was applied to measure functional connectivity of the caudate nucleus, an important “information hub” —the impairment of which is anticipated due to dopaminergic degeneration in the caudate^23^ in Parkinson’s disease. Estimating the spatial pattern of cerebral blood flow (CBF) in pCASL, we can also measure Alzheimer’s disease-like brain activity. We have previously demonstrated that Parkinson’s dementia patients also show Alzheimer’s disease-like whole-brain metabolic/perfusion pattern, but cognitively normal Parkinson’s patients do not^24^.

In this study, we examined Parkinson’s disease-specific and Alzheimer’s disease-like patterns in non-demented Parkinson’s disease patients using resting-state fMRI (i.e., caudate connectivity) and pCASL (Alzheimer’s disease-like perfusion pattern), respectively. Based on the dual syndrome hypothesis, we hypothesized that cognitive performance in Parkinson’s disease patients is independently related to the caudate nucleus connectivity and Alzheimer’s disease-like perfusion pattern score.

## Materials and methods

### Study participants

Eighteen Parkinson’s disease patients (14 male, 4 female; mean age 67.1 ± 7 years; disease duration 8.67 ± 4.9 years; patients were recruited from the Movement Disorder Clinic in Winnipeg, Canada) and ten age-matched healthy controls (2 male, 8 female; mean age 62.8 ± 6 years; recruited in 2017 via community advertisement) were included in this study. All subjects were native English speakers. The diagnosis of an idiopathic Parkinson’s disease was confirmed by movement disorder specialists using diagnostic criteria, defined as the presence of at least two of the cardinal symptoms with improvement after taking levodopa. Based on their MoCA test performance, patients were further divided into groups: (1) with normal cognitive performance (PDNC; MoCA ≥ 26, n = 10) and (2) with low cognitive performance (PDLC; MoCA < 26, n = 8). Disease status of patients was assessed with the Movement Disorder Society—Unified Parkinson’s Rating Scale (MDS-UPDRS^25^). Patients were examined during the on-state of their routine antiparkinsonian medications. Patient exclusion criteria included any previous diagnoses of dementia, or neurological disorders other than Parkinson’s disease, or contraindication to MRI. Patients were administered the Beck Depression Inventory II^26^ to assess for co-occurring mood disturbance^27^.

Healthy controls were verbally screened for any neurological, psychological, or uncontrolled medical disorder, drug/alcohol abuse, history of severe head injury, or contraindication to MRI. Control exclusion criteria included impaired cognition (defined as MoCA score < 26)^28^, or mood disturbance (BDI-II > 10).

This study was approved by the Biomedical Research Ethics Board of University of Manitoba and performed in accordance with its regulations. All participants directly provided written informed consent prior to participating.

### MRI data acquisition

All subjects underwent MRI using a 3 T Siemens/IMRIS MR System equipped with an 18 channel head coil located at the Kleysen Institute for Advanced Medicine at the University of Manitoba. A high resolution T1-weighted image was acquired for anatomical localization using a 3D structural MPRAGE. Resting state functional MRI scanning parameters are as follows: Repetition Time [TR] = 2000 ms; Eco Time [TE] = 28 ms; Flip Angle = 77◦; Slice Thickness = 4 mm; Field of View [FOV] = 220 × 220mm^2^; voxel size = 3.4 X 3.4 X 4.0 mm; scan duration = 11 min. During scanning, subjects were instructed to keep their eyes open and let their mind wander, but not to fall asleep. The CBF acquisition utilized the pCASL pulse sequence with an acquisition time of 5 min. Acquisition parameters were: TR = 4.0 s, TE = 12 ms, FOV = 240 × 240 mm^2^, matrix = 64 × 64 × 20, slice thickness = 5 mm, inter-slice space = 1 mm, labelling time = 2 s, post label delay time = 1.2 s, bandwidth = 3 kHz/pixel, flip angle = 90°. Forty-five label/control image pairs were acquired for each subject.

### Functional connectivity analysis

Standard preprocessing was applied to fMRI data using CONN (https://nitrc.org/projects/conn) running on SPM12 (https://www.fil.ion.ucl.ac.uk/spm/software/spm12/). Resting-state scans were co-registered to participants’ structural T1-MRI scans, spatially normalized to template MRI (MNI space—Montreal Neurological Institute), then smoothed (FWHM = 8 mm × 8 mm × 8 mm). Individuals’ T1 images were segmented and a grey matter probability map was constructed for masking (inclusive) 118 different regions of interest (ROIs) defined by Automated Anatomical Labelling^29^, which additionally include left and right pons^30^. For key subcortical ROIs (i.e., caudate, putamen, pallidum, thalamus, and pons), a cerebrospinal fluid (CSF) map was used for masking (exclusive). For denoising, linear regression was performed with confounding variables of white matter, CSF, realignment, scrubbing, and global signal. Band-pass filter was then applied (0.008–0.09 Hz) and linear detrending performed.

The region-to-region connectivity matrix (z-matrix) was constructed using individually masked ROIs. The z-matrix was sorted, and adjacency matrices were defined with varying cost (1–50%), e.g., at 25% cost threshold, the top 25% of the z-values were set to 1 and the rest were set to 0 excluding the diagonal elements; therefore, the graph was undirected and unweighted, and both positive and negative connectivity have been considered^31^. The minimum cost threshold that resulted in fully connected adjacency matrix for all subjects was 15%. To ensure that our analysis was not dependent on the specific cost thresholding, adjacency matrices were considered at varying cost of 15–25%. Graph theory metrics at each of these costs were averaged together for a mean value measure. These metrics included characteristic path length, clustering coefficient, smallworldness, global and mean local efficiency, and were compared between groups^32,33^. At a regional level (left and right caudate nucleus), betweenness centrality (BC) was also estimated. BC represents if a given node is within the shortest pathway connecting any other pair of nodes. BC identifies nodes that are crucial for information flow in a brain network. In other words, network hubs tend to have high BC values. For graph theory analysis, the Brain Connectivity Toolbox^34^ and in-house programs running on MATLAB 8.3.0 (Mathworks, Inc.) were utilized.

### Alzheimer’s disease-like CBF pattern analysis

The CBF maps were derived from pCASL data as previously described^35^ using ASL Perfusion MRI data processing toolbox (https://cfn.upenn.edu/~zewang/asltbx.php). The resulting CBF images were co-registered to the corresponding T1-weighted image, spatial normalized by wrapping to the MNI standard space, then smoothed using 8 mm Gaussian filter. Preprocessing was done using SPM12 with the default parameters. We then used a classification method to identify Parkinson’s disease patients with Alzheimer’s disease-like CBF pattern expression as previously described^24^. This pattern is characterized by decreased activity in the precuneus, the medial frontal lobes, the temporal lobes, and the cingulum; and relatively increased activity in the somatosensory-motor areas, basal ganglia, thalamus, and cerebellum. If a subject has a high score, their brain metabolic pattern looks more like Alzheimer’s disease patients. We have previously compared the classification accuracy of three different methods, i.e., general linear model, scaled subprofile modelling, and support vector machine (SVM). We showed that SVM-sequential minimal optimization (SMO)-based Alzheimer’s disease classifier was also sensitive to Parkinson’s disease dementia, and that perfusion imaging may be also useful and potentially replacing fluorodeoxyglucose positron emission tomography (FDG-PET)^24^.

### Statistical analysis

Statistical analysis was performed using SPSS (*IBM Corp., Armonk, NY*). Shapiro–Wilk test was performed to determine the normal distribution of each variable. For normally distributed variables (age, AD-like CBF pattern score, characteristic path length, and mean local efficiency), one-way ANOVA was used to assess group differences, followed by post-hoc Bonferroni test if applicable. For variables not normally distributed (sex, caudate BC, disease duration, UPDRS-III, BDI-II, LEDD, clustering coefficient, global efficiency, and smallworldness), Kruskal–Wallis and Mann–Whitney tests were used to determine group differences. Spearman’s correlation was used to examine relationships between imaging-based variables (caudate BC and AD-like CBF pattern score) and MoCA in Parkinson’s disease patients. Results were considered significant at a threshold of *p* < 0.05.

## Results

### Demographic data

The relevant demographics and clinical variables of each group are presented in Table 1. There was no significant difference in age between groups. As expected, PDLC had significantly lower MoCA scores compared to both PDNC and controls (*p* < 0.001, Mann–Whitney U). PDNC and healthy controls were not significantly different in MoCA (*p* = 0.971, Mann–Whitney U). Sex was not equally distributed between groups (more female participants in the healthy control group) (χ2 = 8.08, *p* = 0.012). PDLC had significantly higher BDI-II than controls (*p* = 0.002, Mann–Whitney U), while not significantly different from PDNC (*p* = 0.072, Mann–Whitney U). PDNC and healthy control BDI-II scores were not significantly different from each other (*p* = 0.230, Mann–Whitney U). There was no significant difference between patient groups (PDNC vs. PDLC) in age, sex, disease duration, UPDRS-III motor subscale or LEDD (*p* > 0.3).

**Table 1:** Demographic and clinical variables.

	Healthy control subjects (n = 10)	Parkinson’s disease patients, normal cognition (PDNC; n = 10)	Parkinson’s disease patients, low cognition (PDLC; n = 8)
Age (years)	62.8 ± 6.3	66.5 ± 6.8	67.9 ± 7.6
Male/female^$^	2 M/8F	8 M/2F	6 M/2F
MoCA scores	28 ± 1	28.14 ± 1.2	22.7 ± 1.1***
BDI-II scores	3 ± 3.06	5 ± 3.3	10.7 ± 7.3*
Disease duration (years)	–	9.4 ± 4.9	7.7 ± 4.7
MDS-UPDRS-III	–	20.1 ± 8.4	21.9 ± 10
LEDD total (mg/day)	–	576 ± 698	640 ± 466

Values are listed as mean ± standard deviation.

^$^* p*  < 0.012 by chi-square test.

*** p  < 0.001 by Mann–Whitney in PDLC vs. PDNC and Healthy control.

* p  = 0.002 by Mann–Whitney in PDLC vs. control.

### Global network analysis

Unlike prior studies^36–38^, there were no significant group differences in any of the graph metrics in whole-brain analyses (characteristic path length, clustering coefficient, mean local and global efficiency, and smallworldness) (*p* > 0.1; Table 2).

**Table 2:** No differences in global network analyses between groups.

Global network metrics	Healthy controls (n = 10)	Parkinson’s disease, normal cognition (PDNC; n = 10)	Parkinson’s disease, low cognition (PDLC; n = 8)
Characteristic path length	2.00 ± 0.04	1.97 ± 0.07	1.96 ± 0.03
Clustering coefficient	0.46 ± 0.04	0.44 ± 0.06	0.43 ± 0.02
Global efficiency	0.55 ± 0.01	0.56 ± 0.02	0.56 ± 0.006
Mean local efficiency	0.68 ± 0.02	0.67 ± 0.03	0.67 ± 0.01
Smallworldness	1.10 ± 0.83	1.50 ± 0.70	1.61 ± 0.6

Values are listed as mean ± standard deviation.

### Caudate functional connectivity correlates with cognitive performance in Parkinson’s disease

**Figure 1:**
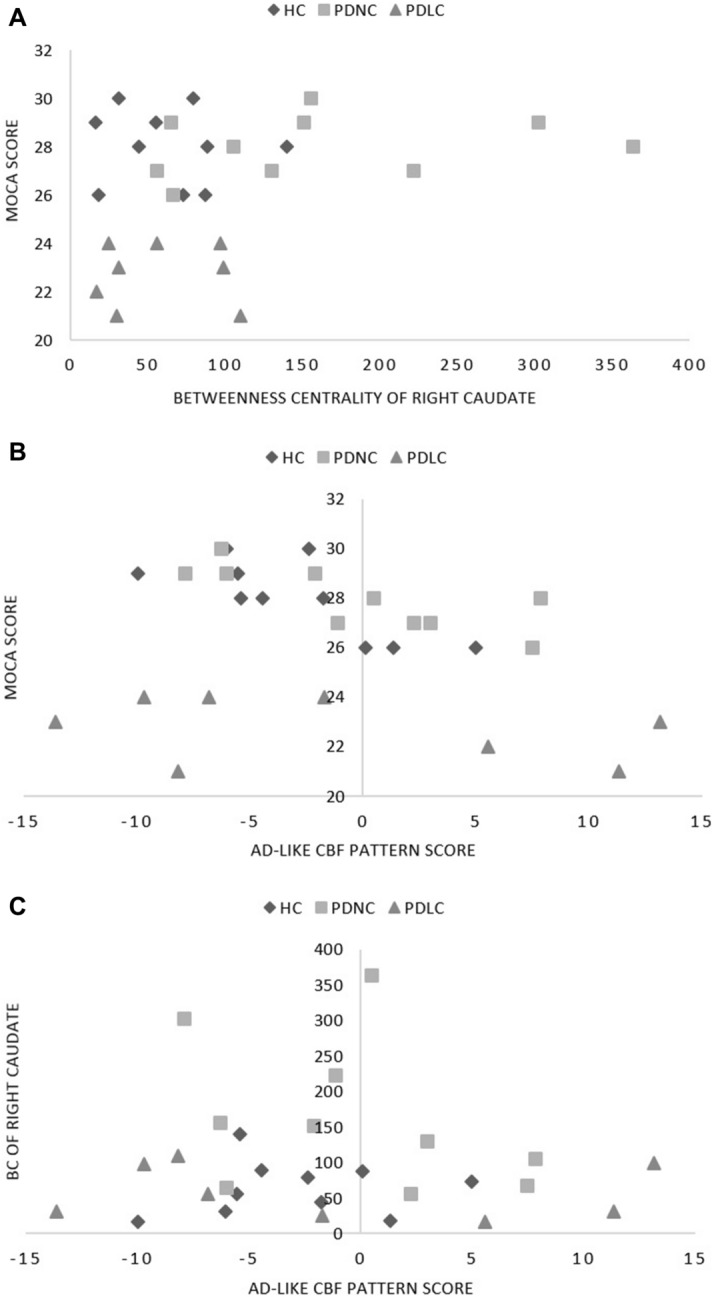
HYPERLINK "sps:id::fig1||locator::gr2" The relationship across caudate connectivity, AD-like CBF pattern, and cognitive performance. (**A**) Right caudate BC and MoCA scores were positively correlated in the Parkinson’s group only (rho = 0.629, *p* = 0.005), and not in the healthy controls (rho =  − 0.19, *p* = 0.59). (**B**) AD-like CBF pattern scores and MoCA scores were negatively correlated in controls (rho =  − 0.782, *p*  = 0.008) but not in PD patients (rho =  − 0.122, *p*  = 0.631). (**C**) No significant correlation between BC of the right caudate and the AD-like CBF pattern scores was found in either group (*p*  > 0.58).

**Figure 2:**
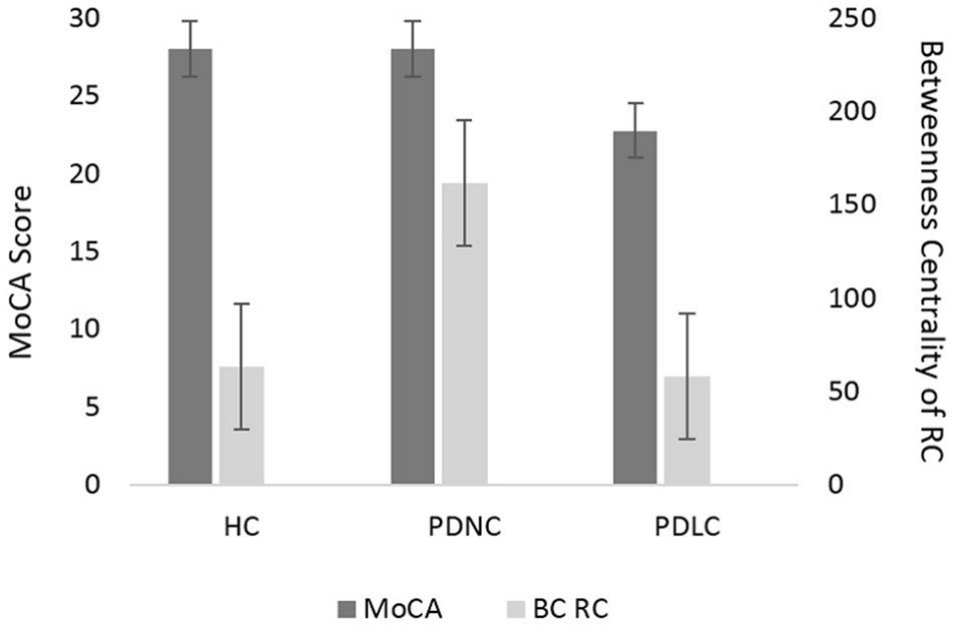
Cognitive performance (MoCA) and right caudate BC. Kruskal–Wallis test showed significant group effects in MoCA score (H(2) = 16.85, *p*  < 0.0005) and right caudate (RC) BC (H(2) = 9.15, *p*  = 0.01). PDLC MoCA scores were lower than both groups (*p  < 0.001, Mann–Whitney test), while PDNC MoCA scores were similar to HC (*p*  = 0.971, Mann–Whitney test). Interestingly, the RC BC of PDNC patients were higher compared to both HC and PDLC (*p*  < 0.011, Mann–Whitney test). Figure represents mean MoCA and BC for each group. Error bars indicate standard error. Results were considered significant at a threshold of *p*  < 0.05.

To examine the role of the caudate nucleus as an information hub, and the effect of its dysfunction in Parkinson’s disease cognitive deficits, we measured correlation between caudate BC and MoCA scores. We found a strong positive correlation between the BC of the right caudate nucleus and MoCA scores in Parkinson’s disease patients (rho = 0.629, *p* = 0.005) but not in healthy controls (rho =  − 0.19, *p* = 0.59; Fig. 1A). Interestingly, PDNC’s BC of the right caudate was significantly different from both PDLC and normal controls (*p* < 0.012, Mann–Whitney), while PDLC and normal subjects were not significantly different from each other (*p* = 0.965, Mann–Whitney; Fig. 2). The left caudate BC was not correlated with MoCA scores in either the Parkinson’s disease group (*p* > 0.9) or healthy controls (*p* > 0.5).

Neither caudate BC variables nor MoCA scores were correlated with age, or medications, within each group. No significant difference between sexes was observed for MoCA and the caudate BC variables within each group (*p* > 0.14). There was no significant correlation between BDI scores and MoCA in Parkinson’s patients (*p* > 0.45). However, BDI scores correlated with MoCA scores in the healthy control group (rho = 0.721, *p* = 0.019).

### Alzheimer’s disease-like CBF pattern expression and caudate betweenness centrality

As expected, Alzheimer’s disease-like CBF pattern score was correlated with MoCA in healthy controls (rho =  − 0.669, *p* = 0.034), suggesting the feasibility of using FDG-PET-based Alzheimer’s disease-like pattern quantification in pCASL-based perfusion imaging. However, the correlation was not observed in patients (rho =  − 0.339, *p* = 0.216; Fig. 1B). No group differences (PDLC vs. PDNC vs. Healthy controls) were observed either (*p* = 0.444, Kruskal–Wallis). No significant correlation was observed between caudate BC and AD-like CBF pattern score (*p* > 0.58, Fig. 1C). This score was not correlated with any other variables, and there were no group differences in AD-like CBF pattern scores (*p* = 0.673, one-way ANOVA).

## Discussion

In this study, we found that BC of the right caudate positively correlated with MoCA scores in Parkinson’s disease. Interestingly, the PDNC group showed significantly higher BC scores for the right caudate than both PDLC and normal controls. We also found the Alzheimer’s disease-like CBF pattern score was not correlated with MoCA scores in Parkinson’s disease.

The positive correlation between the BC of the right caudate and MoCA scores supports the current literature claiming the caudate is involved with Parkinson’s disease cognitive deficits^12,39,40^. To reiterate, the dual syndrome hypothesis posits the underlying dysfunction of cognitive impairment in non-demented Parkinson’s disease is in the frontostriatal circuit involving the caudate nucleus and prefrontal cortex. Our patients’ cognitive status is mildly impaired (i.e., PDLC), and the correlation that we have found between the BC of the caudate and MoCA scores further supports this hypothesis.

The association between cognitive performance and caudate BC was only seen in the right caudate nucleus, not the left. Laterality of the caudate in Parkinson’s disease has been reported in many studies, using different imaging modalities. With PET imaging, an association between the right caudate and cognitive performance has been reported: the authors found decreased dopaminergic function of the right caudate was related to slow processing time, using the Stroop test in patients at an early stage of Parkinson’s disease^41^. More prominent hypometabolism in the right caudate has been shown in cognitively impaired Parkinson’s disease patients^42^. This may suggest that the right caudate plays a more crucial role in maintaining cognitive functions (or at least the subdomain of cognitive functions that are more accurately assessed by MoCA), and its deterioration could lead to more cognitive impairment.

More detailed group analysis revealed PDNC had higher right caudate BC than both PDLC and controls, and similar MoCA scores to controls. We suggest this increase in “hubness”^43^ to be a successful compensatory mechanism, for maintaining cognitive performance in PDNC. Similarly, Pereira and colleagues^44^ demonstrated an increase in frontal hubs in PDNC, compared to both healthy controls and Parkinson’s disease with MCI. While it is difficult to assess the causality for this shifted frontal connectivity, it should be noted that the caudate receives dense projections from the dorsolateral prefrontal cortex^45^, which directly controls dopamine release in the caudate^46,47^. Nevertheless, since BC is a relative measure, it is also possible that the increased BC is a result of the relative maintenance of frontal area connections compared to degenerative areas in early Parkinson’s disease.

Caudate dopamine degeneration has been associated with cortical hypometabolism characterized as Parkinson’s disease cognition-related metabolic pattern (PDCP)^48^, which does not emerge until after 2 years from the initial diagnosis but slowly progresses over the years^49^. This suggests that the prefrontal-caudate circuitry is relatively intact in early stage Parkinson’s disease although the overall dopamine level in the caudate nucleus is already significantly lower than age-matched normal subjects^50^. The capacity for compensation in the remaining dopaminergic neurons has already been established^51^. The more spatially diffused dopaminergic innervation in the striatum^52,53^ may be associated with increased functional connectivity. Increased resting-state connectivity within the striatum, particularly the caudate, has previously been suggested to aid in cognitive maintenance in Parkinson's disease^54^. Further, no age-related changes were noted in caudate BC in our healthy control individuals (n = 37, unpublished observation). This suggests the high caudate BC is a unique feature to PDNC. A minority of Parkinson’s patients (approximately 20%) with a disease duration reaching 8 years do not go on to develop dementia^55^. Our PDNC cohort had an average disease duration of 9.4 ± 2.6 years. Their success in maintaining normal cognition may therefore be associated with their high levels of caudate BC.

According to the dual syndrome hypothesis, another important axis of Parkinson’s cognitive deficits is the posterior cortical and temporal abnormality—traditionally thought to be relevant to Alzheimer’s disease-related pathology^14^. FDG-PET is the most frequently used imaging method that complements diagnosis of Alzheimer’s disease, and is often used to quantitate disease progression^56^ and treatment responses^57^. We recently developed and validated automated quantification methodology to estimate how likely an individual FDG-PET scan belongs to an Alzheimer’s disease patient^24^. Results suggested that this SVM-SMO-based method was sensitive to Parkinson’s disease dementia as well, but not to cognitively normal Parkinson’s disease patients.

MoCA and Alzheimer’s disease-like CBF pattern scores were not correlated in Parkinson’s disease groups. One potential explanation is that cognitive impairment in our patients (without dementia) was primarily driven by frontostriatal abnormality, and less from Alzheimer’s disease-like pathology. This negative observation further aligns with the dual syndrome hypothesis^14^: demented patients’ pathology is more similar to Alzheimer’s, differing from MCI pathology. A carefully designed longitudinal study is warranted to confirm this hypothesis.

We did not identify any significant differences in global graph theory metrics between the groups, posing a potential limitation to this study. Metrics such as increased clustering coefficient^58^, and characteristic path length with reduced global efficiency^44^ in Parkinson’s MCI compared to non-demented Parkinson’s disease and healthy controls have been previously reported. However, other studies have reported maintained global integration in Parkinson’s disease compared to healthy controls^30,59^. Most of these studies were done on drug-naïve Parkinson’s disease patients, or while off medication. Our patients in this study were examined while on their prescribed medications. This raises another limitation—the impact of antiparkinsonian medication on perfusion has been previously documented^60^. Studies suggest chronic levodopa treatment may induce angiogenesis and increase vascular sensitivity in the putamen^61,62^. It is less evident if the caudate is also involved in this hyper-vascularity phenomenon, which may have influenced its BC measurement. It is also unknown if the Alzheimer’s disease-like pattern score estimation using pCASL MRI is influenced by the use of antiparkinsonian medications.

While MoCA provides insightful information about patients’ cognitive status, it is our limitation that PDLC vs. PDNC was primarily determined using only MoCA scores, and not by thorough neuropsychological exams. Different types of assessments were used to screen dementia in our patients (e.g., DSM-4 vs. DSM-5 criteria), and specific results for assessments other than MoCA were not disclosed, as the data was not de-identified. Nevertheless, it should be noted that our primary outcome is the correlation between MoCA and caudate BC in Parkinson’s disease as one group, and the division between PDLC and PDNC was only done in a post-hoc manner to understand the effect of cognitive status in relation to healthy control subjects.

Other constraints include small sample sizes for each group. This limited further subgroup analyses, involving handedness and most-affected hemisphere (motor symptoms). When included as a covariate, sex and BDI-II scores did not significantly influence results. Nevertheless, given the small group sizes, and their disparity in sex and BDI-II scores, these results should be interpreted with caution, as a preliminary investigation of neural compensatory mechanisms for PD patients.

## Conclusions

Our findings support and complement the dual syndrome hypothesis, i.e., Parkinson’s disease executive dysfunction is more related with dopaminergic pathways (prefronto–caudate) and visuospatial dysfunction is more related with cholinergic (parieto–temporal); the latter being more relevant for dementia, while the former is more involved with MCI. Our study suggests these two pathologies are dissociated, and increased network hubness of the right caudate nucleus may potentially involve a compensatory mechanism.

## Data Availability

The raw data are not publicly available as they contain patient medical data which can only be accessed under the Personal Health Information Act (PHIA). Information regarding this can be found at https://www.gov.mb.ca/health/phia/.

## Abbreviations

AD: Alzheimer’s disease
BC: Betweenness centrality
BDI-II: Beck depression inventory test. *Note:* BDI scores were unavailable for 3 PDNC patients.
CBF: Cerebral blood flow
HC: Healthy controls (n = 10)
LEDD: Levodopa equivalent daily dose
MDS-UPDRS-III: Movement disorder society—unified Parkinson’s disease rating scale—III
MoCA: Montreal cognitive assessment test
PD: Parkinson’s disease group.
PDLC: Parkinson’s disease patients with low cognitive performance measured by MoCA test (n = 8)
PDNC: Parkinson’s disease patients with normal cognitive performance measured by MoCA (n = 10)
RC: Right caudate
